# Development and preliminary validation of a post-fistula repair reintegration instrument among Ugandan women

**DOI:** 10.1186/s12978-017-0372-8

**Published:** 2017-09-02

**Authors:** Alison El Ayadi, Hadija Nalubwama, Justus Barageine, Torsten B. Neilands, Susan Obore, Josaphat Byamugisha, Othman Kakaire, Haruna Mwanje, Abner Korn, Felicia Lester, Suellen Miller

**Affiliations:** 1 Bixby Center for Global Reproductive Health, Department of Obstetrics, Gynecology and Reproductive Sciences, University of California, San Francisco, USA; 2 Department of Obstetrics and Gynaecology, Makerere University College of Health Sciences, Kampala, Uganda; 3 Urogynecology Division, Mulago National Referral and Teaching Hospital, Kampala, Uganda; 4 Center for AIDS Prevention Studies, Division of Prevention Sciences, Department of Medicine, University of California, San Francisco, USA

**Keywords:** Obstetric fistula, Social reintegration, Maternal morbidity, Obstructed labor, Measure, Instrument, Uganda

## Abstract

**Background:**

Obstetric fistula is a debilitating and traumatic birth injury affecting 2–3 million women globally, mostly in sub-Saharan Africa and Asia. Affected women suffer physically, psychologically and socioeconomically. International efforts have increased access to surgical treatment, yet attention to a holistic outcome of post-surgical rehabilitation is nascent. We sought to develop and pilot test a measurement instrument to assess post-surgical family and community reintegration.

**Methods:**

We conducted an exploratory sequential mixed-methods study, beginning with 16 in-depth interviews and four focus group discussions with 17 women who underwent fistula surgery within two previous years to inform measure development. The draft instrument was validated in a longitudinal cohort of 60 women recovering from fistula surgery. Qualitative data were analyzed through thematic analysis. Socio-demographic characteristics were described using one-way frequency tables. We used exploratory factor analysis to determine the latent structure of the scale, then tested the fit of a single higher-order latent factor. We evaluated internal consistency and temporal stability reliability through Raykov’s *ρ* and Pearson’s correlation coefficient, respectively. We estimated a series of linear regression models to explore associations between the standardized reintegration measure and validated scales representing theoretically related constructs.

**Results:**

Themes central to women’s experiences following surgery included resuming mobility, increasing social interaction, improved self-esteem, reduction of internalized stigma, resuming work, meeting their own needs and the needs of dependents, meeting other expected and desired roles, and negotiating larger life issues. We expanded the Return to Normal Living Index to reflect these themes. Exploratory factor analysis suggested a four-factor structure, titled ‘Mobility and social engagement’, ‘Meeting family needs’, ‘Comfort with relationships’, and ‘General life satisfaction’, and goodness of fit statistics supported a higher-order latent variable of ‘Reintegration.’ Reintegration score correlated significantly with quality of life, depression, self-esteem, stigma, and social support in theoretically expected directions.

**Conclusion:**

As more women undergo surgical treatment for obstetric fistula, attention to the post-repair period is imperative. This preliminary validation of a reintegration instrument represents a first step toward improving measurement of post-surgical reintegration and has important implications for the evidence base of post-surgical reintegration epidemiology and the development and evaluation of fistula programming.

## Plain English summary

Obstetric fistula is a debilitating and traumatic birth injury affecting 2–3 million women globally, mostly in sub-Saharan Africa and Asia. Women who develop fistula suffer from poor physical and mental health, and have economic concerns. Access to surgical treatment is increasing for women affected by fistula, but not enough attention is paid to women’s physical and mental health following surgery. Our study sought to develop and test a measure of women’s return to normal life following fistula surgery through speaking with women about their experiences and following women’s recovery experiences for one year. Women’s main concerns following surgery were resuming mobility, increasing social interaction, improving self-esteem, starting to work again, and meeting their own needs and the needs of their dependents, meeting other expected and desired roles, and negotiating larger life issues. We revised an existing measure to better represent these themes and the context of our population. Analysis of this measure found that the reintegration concept was made up of four main themes: ‘Mobility and social engagement’, ‘Meeting family needs’, ‘Comfort with relationships’, and ‘General life satisfaction’, and that these four themes fit within a higher factor of ‘Reintegration.’ Women’s reintegration scores were compared to their reports of quality of life, depression, self-esteem, stigma, and social support. The measure that we developed may be used for improving our understanding of the time following fistula surgery and in the evaluation of fistula programs.

## Background

Obstetric fistula is a debilitating maternal birth trauma primarily caused by prolonged obstructed labor compounded by delays in accessing comprehensive emergency obstetric care. While robust data on the incidence and prevalence of obstetric fistula do not exist due to measurement challenges [1–3], available community and facility-based data support obstetric fistula as an important public health concern. Estimates suggest as many as 2 million women globally may be afflicted, the majority in sub-Saharan Africa and Asia, with between 50,000 to 100,000 annual incident cases [4, 5].

Obstetric fistula is associated with a variety of severe physical, psychological and social sequelae [6–10], referred to as ‘obstructed labor injury complex’ to acknowledge the many pathologies endured across multiple organ systems [11]. Urinary and/or fecal incontinence is typically the primary presenting clinical symptom of this condition; however, neurologic injury, gynecologic morbidity and orthopedic trauma are very common, resulting in infections, vaginal and genital ulcerations, difficulty walking without assistance, and secondary infertility, among others. The psychological and social sequelae are equally devastating; consistent leakage of urine and/or feces results in affected women and girls being stigmatized and ashamed of their offensive smell and inability to remain clean which often leads to isolation from families and communities [12, 13].

Surgical treatment of obstetric fistula results in clinical success rates ranging from 65% – 95% [14, 15]. Available at specialized treatment centers, efforts by local, national and international agencies have dramatically improved women’s access to obstetric fistula surgery over the past decade through expanded specialized surgical training and covering medical costs. Approximately 23 thousand fistula surgeries occurred globally in 2014–2015 [16]. However, previous research on the success of fistula surgery is largely limited to the short-term clinical outcomes of surgery, largely assuming concomitant positive social and emotional effects with successful surgery [7, 17, 18]. While clinical fistula closure is an important indicator for tracking surgical outcomes, given the vast array of physical and psychosocial correlates of fistula, it may not represent an adequate indicator of recovery.

Only recently have researchers begun longitudinal assessment of post-surgical recovery processes after fistula repair to better understand women’s experiences. General improvements in perceived quality of life, particularly among women with successful surgery, have been reported [19, 20]. However, multiple studies have documented challenges, including persistent post-repair fistula-related symptoms such as residual incontinence [18, 21, 22], pain and weakness [19, 23], and sexual and fertility complications [9, 19, 24–26]. Women experiencing lingering physical problems such as these are less able to resume their previous roles and less likely to consider themselves recovered [7, 19, 20, 27]. These women are at greater risk of poor continued mental health [23, 28, 29]. For all women, anxieties around fistula recurrence have been reported as contributors to low mental health status and as affecting women’s resumption of sexual activity and intimate relationships [19, 23, 25, 30, 31]. Further studies have highlighted the positive influence of returning to work and economic productivity in the reintegration process [31, 32].

Extension of fistula care to include robust post-surgical rehabilitation efforts that meet the continuing needs of women beyond surgery is necessary to improve recovery from a holistic perspective. Furthering the evidence base around reintegration following obstetric fistula surgery and understanding the effect of potential interventions to enhance the reintegration experience requires an explicit attention to measurement. However, currently no standardized measurement tool exists to evaluate how successful a woman’s post-surgical reintegration has been. Therefore, the primary goal of this study was to develop and pilot test a measurement instrument to assess the level of reintegration among women following obstetric fistula surgery. The development of such a tool will not only contribute to knowledge of the epidemiology of reintegration, but also has important implications for the development and evaluation of evidence-based programming for the growing population of women with fistula repair.

## Methods

We conducted an exploratory sequential mixed-methods study, qualitative followed by a quantitative component, to develop and validate a measure evaluating family and community reintegration following obstetric fistula surgery [33]. The target population for our study was women accessing obstetric fistula surgery at Mulago Hospital in Kampala, Uganda. Mulago Hospital is the national referral and teaching hospital for Makerere University College of Health Sciences. Fistula repair is provided by the urogynecology division as both an ongoing surgical service and supplemented by four to five annual fistula repair camps.

### Qualitative component

We first conducted formative research to understand the domains most important for social reintegration among women who had been surgically treated for obstetric fistula (June – August 2014). Women were eligible for participation in this phase of the study if they had undergone obstetric fistula surgery within the previous 6–24 months at Mulago Hospital, spoke Luganda or English, resided within 100 km of Mulago Hospital, provided a telephone contact at surgery, and were able to provide informed consent for study participation. We developed a list of patients meeting the time, residence and telephone requirements of our eligibility criteria and we contacted and screened these individuals by telephone. Of the 45 eligible women contacted, most (44) provided verbal consent for participation. 33 ultimately participated; 11 did not participate due to achievement of data saturation.

Research staff conducted in-person written informed consent procedures with all participants. Study staff administered a short quantitative survey to all participants to capture the following characteristics: age, tribe, religion, household socio-economic status, marital status and history, employment and financial status, educational attainment, pregnancy and obstetric history, general health and level of continence, and receipt of post-surgical counseling. Level of continence was evaluated with the International Consultation on Incontinence Questionnaire Short Form [34], and general health was evaluated with the Stanford Self-Rated Health measure [35]. Participants subsequently participated in semi-structured in-depth interviews (*n* = 16) or focus group discussions (*n* = 17; 4 groups); in-depth interviews sought to elicit each woman’s experience developing and living with fistula, the processes they underwent seeking fistula treatment, and their experiences with surgery and reintegrating to their families and communities after fistula repair.

Focus group discussions were targeted toward exploring reintegration following fistula repair. We operationalized the concept of reintegration as the experience of healing from the fistula and getting life back to ‘normal’ after repair surgery. Interviews and focus groups were conducted in Luganda language in a private office within the urogynecology department at Mulago Hospital. On average, interviews and focus group discussions lasted 1 and 1.5 h, respectively. Participants were reimbursed for their round-trip transportation expenses and provided with refreshments. In-depth interviews and focus group discussions were audio recorded with the explicit permission of participants and concurrently translated and transcribed into English for analysis by an experienced translator. Audio recordings were archived following transcription.

Univariate analyses were performed to describe the socio-demographic characteristics of participants through calculating means and standard deviations for continuous variables and proportions across categorical variables in Stata v14 software (StataCorp, College Station, TX). Transcripts from in-depth interviews and focus group discussions were coded using inductive and deductive codes within Atlas.ti software and analyzed for domains most relevant to family and community integration by two researchers, one Ugandan and one American. Coding disagreements were resolved by discussion. Thematic analysis was concurrent with data collection to assist in determining achievement of data saturation [36]. Coded data were analyzed thematically to describe the different dimensions and commonalities of each theme and the patterns and linkages between themes.

### Literature review and instrument development

Concurrent with our qualitative work, we reviewed the literature in order to identify a selection of validated measures developed to capture constructs of rehabilitation or reintegration among different populations. Based on this review, we critically evaluated the following tools: the Barthel Index of Activities of Daily Living [37], the Community Integration Questionnaire [38, 39], the Community Integration Measure [40], the Reintegration to Normal Living Index (RNLI) [41, 42], and the Sydney Psychosocial Reintegration Scale [43, 44]. Of the five measures, the Reintegration to Normal Living Index, which focuses on the global function of a patient during rehabilitation and that patient’s ability to return to normal life, touched on the majority of the constructs that were identified from our formative work. Furthermore, it had previously been successfully utilized among women recovering from obstetric fistula in Tanzania [31]. However, the language and item coverage were not completely congruent with our formative research and population (e.g., the RNLI was developed for populations living in non-agricultural western societies, and did not address different relationship types, etc.). We modified and extended the 11 items in the RNLI to a 22-item scale that addressed the topics that had emerged as most important during our qualitative work: mobility: house, community, longer trips; comfort with meeting self-care needs; work: engagement in work, satisfaction with capacity to work and meet needs of self/dependents; participation in social and religious activities; family role meeting individual and family needs; satisfied with community role; comfort with relationships; and satisfaction with self, life, and health.

After review by key stakeholders, including individuals working with women affected by obstetric fistula and the women themselves, we followed the WHO recommended process for translation and adaptation of instruments: forward translation, expert panel back translation; pre-testing and cognitive interviewing [45]; and ultimately finalizing the measure for pilot testing within a longitudinal cohort of 60 women followed for 12-months post-surgery (Appendix).

### Quantitative component

We recruited a longitudinal cohort of women at the time of obstetric fistula surgery in order to quantitatively validate the draft instrument to measure level of reintegration. Women were eligible for participation in the longitudinal cohort upon completion of initial examination and clearance for obstetric fistula surgery at Mulago if they spoke Luganda or English, resided in a community with cellular telephone coverage, and were able to provide informed consent for study participation. Patients confirmed for surgery were approached and screened for eligibility by the research staff, who obtained written informed consent from those who were interested. Where eligible participants were unable to be approached prior to surgery, they were asked to participate post-operatively and following sufficient recovery to be able to converse with the research staff. Participants were recruited from December 2014 to June 2015. A sample size of 60 was targeted based on feasibility. Data were captured from our longitudinal cohort participants at baseline, 2 weeks, and 3, 6, 9 and 12-months post-surgery. Our baseline questionnaire was administered in person by the local research coordinator and included basic socio-demographic questions, the newly developed measurement tool, and mental health, quality of life, self-esteem, trauma and social support measures. We included the following validated measurement tools: the International Consultation on Incontinence Questionnaire Short Form (ICIQ-SF) [34], the Stanford Self-Rated Health measure for general health [35], the Hopkins Symptom Checklist for depression [46, 47], the WHO QOL BREF for quality of life [48, 49], the Rosenberg self-esteem scale [50], a modified version of the HIV/AIDS Stigma Instrument [51], the Primary Care PTSD Screen and the Brief Trauma Questionnaire for trauma [52, 53], and the multidimensional scale of perceived social support [54, 55]. Two-week data collection was conducted prior to participant discharge from the hospital, and included only the newly developed measurement instrument for evaluation of temporal stability reliability. All other follow-up surveys queried around modifiable socio-demographic questions, the reintegration measurement tool, the validated measures listed above, and physical symptoms. All four post-discharge follow-up surveys were administered over mobile telephone by the research staff, as participants were provided with cell phones and monthly airtime contracts for the duration of the follow-up. Data for the current analyses were limited to the baseline and two-week collection period as data capture at baseline was complete for our full sample and the availability of the measure at two weeks. As the construct of reintegration is a condition-specific measure of global functional status, measurement at baseline was deemed acceptable for purposes of preliminary validation.

We evaluated the validity and reliability of the measure as follows: Content validity was established through review by key stakeholders and clinical and research experts (2 nurses and 4 doctors employed in the urogynecology ward at Mulago Hospital). We subsequently conducted cognitive interviewing to pre-test the new instrument among four women seeking surgery for obstetric fistula, and their opinions were used to refine the tool further to suit the needs of the target population. The final instrument was approved by all reviewers before implementation within the longitudinal cohort. Construct validity was examined through known-groups validation by several functional variables considered to be theoretically related to the measure including physical symptoms, mental health concerns, etc. We employed exploratory factor analysis methods to explore the latent variable structure of the scale.

The socio-demographic characteristics and clinical outcomes of the women were described using one-way frequency tables as well as appropriate distributional measures for each variable. Exploratory factor analysis was conducted on the expectation-maximization covariance matrix to explore the latent structure of the scale while accommodating some missingness within the two items which were not universal (i.e., were not applicable to all participants) but which were deemed extremely important for inclusion from a conceptual standpoint: 1) satisfaction with relationship with partner, and 2) satisfaction with relationship with children [56]. We specified a promax oblique rotation to allow for correlation across factors, as was anticipated theoretically, and established a minimum factor loading criteria of 0.3 [57]. We subsequently explored the fit of a single higher-order latent factor to the four-factor structure identified through our preliminary analysis through factor analysis of the factor correlation matrix, again specifying promax oblique rotation. The higher-order factor analysis was then refitted using confirmatory factor analysis (CFA) in order to obtain goodness-of-fit statistics to evaluate the fit of the higher-order factor model to the data. The fit statistics were: the likelihood ratio chi-square test of exact fit, the comparative fit index (CFI), the root mean square error of approximation (RMSEA), and the standardized root mean square residual (SRMR). CFI ≥ .95, RMSEA ≤ .06, and SRMR ≤.08 indicated satisfactory fit of the higher-order factor model [58]. For each subscale extracted from factor analyses and for the single higher-order factor identified, internal consistency reliability was evaluated through Raykov’s *ρ* statistic [59], while temporal stability reliability was evaluated through Pearson’s correlation coefficient at baseline through two weeks (hospital entrance to exit, approximately 10–14 days). Finally, we estimated a series of linear regression models to explore the associations between the standardized reintegration measure (0–100) and validated scales representing theoretically related constructs. All statistical analysis was conducted within Stata v14 (StataCorp, College Station, TX), and differences were considered statistically significant at *p* < 0.05.

The study protocol was approved by the Makerere University College of Health Sciences Research and Ethics Committee (Ref# 2014–052) and the University of California, San Francisco Human Research Protection Program, Committee on Human Research (IRB# 12–09573). All individuals eligible for the research underwent an informed consent process; those individuals unable to provide signature for informed consent provided thumbprint confirmation.

## Results

### Qualitative component

Characteristics of qualitative participants are presented in Table 1. Median age at interview was 30 years while median age at fistula development was 22 years. Time lived with fistula varied, with the majority of women having lived with the condition for at least one year (87%) and 42% having lived with it for more than five years. The majority of participants lived with their husbands (76%).Two-thirds of participants had not completed primary school (63%) and reported being financially dependent upon their husbands (64%). The median number of living children was two; 46% had no living children (not shown).

**Table 1:** Baseline characteristics of study participants across qualitative and quantitative study phases

	Qualitative Participants		Quantitative Participants	
	*n* = 33		*n* = 60	
	n	%	n	%
Current Age (median, IQR)	30 (24–37)		28 (21–36)	
Age at Fistula (median, IQR)	22.5 (18–28)		22.5 (18–31)	
Time Lived with Fistula				
< 1 Month	0	0.0	8	13.3
1–3 Months	0	0.0	19	31.7
3–12 Months	4	12.1	8	13.3
1–2 Years	13	39.4	3	5.0
3–5 Years	2	6.1	5	8.3
> 5 Years	14	42.4	17	28.3
Infant Survival, Fistula-Related Delivery	10	30.3	17	28.3
District				
Kampala	3	9.1	10	16.7
Luweero	4	12.1	3	5.0
Mityana	1	3.0	5	8.3
Mubende	5	15.2	6	10.0
Mukono	2	6.1	4	6.7
Wakiso	8	24.2	18	30.0
Other^a^	10	30.3	14	23.3
Living Situation				
Alone^b^	3	9.1	13	31.6
Husband^b^	25	75.8	24	40.0
Adult children^b^	1	3.0	4	6.7
Parents^b^	0	0.0	8	13.3
Other^b^	4	13.2	11	18.4
Living Children (median, IQR)	2 (1–3)		1 (0–3)	
Educational Attainment				
None	2	6.1	10	16.7
Some Primary	19	57.6	24	40.0
Completed Primary	4	12.1	17	28.3
Any Secondary	8	24.2	9	15.0
Occupation				
None	11	33.3	35	58.3
Vendor/Shopkeeper	4	12.1	5	8.3
Farmer	11	33.3	15	25.0
Other	7	21.3	5	8.3
Primary Source Financial Support				
Self	11	33.3	18	30.0
Husband	21	63.6	21	35.0
Other	1	3.0	21	35.0
Household Assets				
Piped Water	5	15.2	9	15.0
Flush/pour flush toilet	2	6.1	4	6.7
Electricity	10	30.3	26	43.3
Radio	29	87.9	35	58.3
Television	13	39.4	17	28.3
Mobile phone	29	87.9	39	65.0
Refrigerator	3	9.1	26	43.3

^a^ maximum 2 participants per each ‘other’ district; ^b^ with or without young children; iqr = interquartile range

Our qualitative participants described experiences with obstetric fistula characterized by significant suffering, both physically and psychologically. In addition to urinary incontinence, the reported physical sequelae of obstetric fistula included pain, weakness, and infection, all resulting in limited physical mobility. They had substantial self-care needs and were highly dependent on caregivers. Psychologically there was significant evidence of depression, anxiety, and low self-esteem. Stigma was a common experience, generated from varied sources; internalization of stigma frequently led to self-isolation. Disclosure avoidance was a common strategy for evading stigma anticipations. Many participants also highlighted the negative impact that their fistula had on their children, partners, and other family members.

Perceptions of recovery focused largely on achievement of continence, as this was the primary symptom that women associated with the fistula. Participants whose surgery resulted in attainment of urinary continence were exceedingly pleased with the results, and often expressed disbelief that they had healed. One participant exclaimed, “*I felt so good. I would wake up at night and touch around to feel if there was any urine [on the mattress] and indeed there wasn’t!” (22-year old interviewee).*

Themes that emerged as central to women’s experiences following achievement of continence, their perceptions of recovery from the fistula, and ultimately reintegrating included overcoming limited mobility and social participation, improving self-worth and reduction of internalized stigma, resuming work and reducing economic dependence, achieving the ability to meet other expected and desired roles, and changing their overall outlook to being able to consider their lives more broadly.

#### Limited mobility and social participation

During the time they were suffering from fistula, the majority of qualitative participants reported frustration with constraints on their mobility, imposed by the logistics of managing their incontinence, physical pain or discomfort, and stigma anticipations. Limitations on mobility manifested most simply as not having the freedom to do what one wanted, as evidenced by the following:

*"You can’t have peace at any one time, clothes will be burning your skin. You can’t even go to the shop because as you move, urine can pass out and you become wet so you become scared of even visiting your friends, you always keep at home… I couldn’t go anywhere." (19-year-old focus group participant).*

*"I could no longer do anything on my own…. I wasn't moving. I couldn't even move out of my bedroom. There were times when I felt like going out of my bedroom and going out to my neighbors and conversing but it was impossible because you would be standing and you would all get wet." (32-year-old interviewee).*

Elation at being able to move freely following a successful surgery was commonly reported, described by one interviewee: “*I felt so excited and wanted to move all the time! What would hurt me most [living with fistula was] that I couldn’t move…. I can now move freely! [After surgery], I would go to the road side just to feel what I was missing before.” (26-year-old interviewee).*

Frustrations with restricted mobility were also inextricably linked to limitations on opportunities and possibilities for social participation. Women largely reported being unable to freely spend time with family members and friends during the time they lived with fistula, and that this inability to participate socially represented a significant cause of stress, as expressed in the following quotations:

*"The main reason why I am sad is that before I would interact and converse with fellow women unlike now; I can go to them and only interact with them for a short time and leave because of the leaking." (28-year-old interviewee).*

*"Before, I could play, I could mix with people. I could visit friends, grandparents, aunties, but [when I had fistula] I couldn't because I was avoiding the condition of sitting in a place and leaving it wet and I wouldn’t want to reveal this condition to everyone." (19-year old interviewee).*

Resumption of social engagement was consistently mentioned as being very important to women following successful surgery. Sentiments shared by several participants highlighted the social support that social engagement facilitated and the recovered sense of belonging with others, contrary to the alienation they had experienced during the time they suffered from fistula-related incontinence. These sentiments are expressed in the following quotations:

*"Now I have hope because I feel good, I can go anywhere and I also enjoy life because I can be with my friends and even my family which keeps me happy." (19-year old interviewee).*

*“From the time I was operated on, there is nothing that can stop me from going to interact with people; and remember, if you are to develop, you need to relate with people. So from the fact that I am no longer suffering from fistula, I relate with people because I now go and work - whatever happened, happened, but all in all I can meet my basic needs. (37-year old interviewee).”*

*"Now you easily sit beside your friend when they have invited you somewhere but back [when you had fistula], you would sit and you yourself you could feel the cloth you used to pamper yourself really smelling. Now you can sit in a line with people and you feel like a human being too."(33-year old focus group participant).*

#### Self-worth and internalized stigma

Perceptions of recovery heavily weighed on individual’s opinions of themselves and their value. As demonstrated by the following quotes, many participants reported extremely low self-esteem during the period that they were experiencing fistula-related symptoms, perceiving themselves as not worthy of being with others and belonging to their communities.

*"You can’t have fistula and consider yourself to have value; my heart was depressed. For 30 years, I wasn’t considering myself to be a human being." (50-year old focus group participant).*

*"[When I had fistula], I wasn’t considering myself as part of the human race. [I thought of myself as] someone who can’t be among people, someone who can’t fit anywhere; you just regard yourself as something without meaning." (23-year old focus group participant).*

Conversely, after successful surgery, these perceptions of low-value reversed for many, with women largely reporting feeling that they are useful and needed by their families and communities. This sentiment was reflected by one interviewee: *“Now I see myself as a powerful woman with visions and someone who is useful and I see that the community needs me now.” (39-year old interviewee).*

#### Resumption of work and reduced economic dependence

The ability to work played a distinct role in altering women’s perceptions of their self-value through their ability to participate in their previous activities, achieving their previous roles, and through empowering them to meet their own needs and the needs of their dependents. Participants’ narratives frequently compared their ability to work and what this meant to them during the time they lived with significant fistula-related symptoms to the time following surgery:

*"[Initially] I had lost interest, but right now I can even cultivate sweet potatoes. I can now cultivate food which is enough for my children so that they don’t live a needy life apart from need for money. Food, I can offer that very well to my children, they can eat and all be happy. Now I am useful to my home." (47-year old focus group participant).*

*"My roles were regained [after fistula repair]. Initially I could do nothing on my own because of the pain, but now I can do [all of my previous tasks]…. I clean my home and take care of my children like I did initially."(23-year old focus group participant).*

#### Inability to meet other expected roles

Dissatisfaction with being economically dependent on others was not the only cause of tension. Some women described tension stemming from the disconnect between their household caretaking structure due to fistula as the antithesis of normal expectations, both those they held for themselves and those held by their families:

*“I would feel sad from the very inside of me because I would imagine that instead of me helping my husband or mother-in-law; he or she was the one helping me.” (20-year-old interviewee).*

*"I would imagine, my husband was expecting me to bear for him a child but all was in vain because our baby had passed away yet if that baby was alive, we would be loving her so much." (20-year-old interviewee).*

Much of this tension was internal; however, some women shared that it affected the quality of their relationships with important people in their lives.

*“[After the surgery], there was a very big difference with my family relationships; my mother even started liking me more … because I was not giving her anything [during the time I was suffering from fistula]; instead I was milking her, yet [as my mother], she should have been at the receiving end. So our love revived after.” (22-year-old interviewee).*

One woman highlighted the difference in how she is perceived after healing from fistula, describing how she is now able to resuming the role of contributor within her community, “*Even me I see a change because people who couldn’t even associate with me can now ask for, say, sauce in case they don’t have and I am having.” (40-year-old focus group participant).*

#### Overall outlook

Finally, during the time that they were suffering from fistula-related symptoms, women described an inability to look forward, to consider their lives in larger perspective, and how this changed following recovery. One woman shared, *“Then we were only thinking of how to get a polythene [sheet to protect from urine leakage] or transport to take you to a health facility or even money for surgery …. Now everything that comes up we also think of it, like saving money for new things … we can also think of others; say I have 1,000USh let me give my friend 200USh and we share.” (30-year old focus group participant).*

### Quantitative component

Exploratory factor analysis of the baseline data was conducted using oblique rotation. Three of our initial items (satisfaction with how self-care needs are met; feeling down, depressed or hopeless; and feeling hope) did not load on any of these four factors at the minimum level we established for factor loadings, thus were excluded from all further analysis of the scale. Our results suggested a four factor structure (Table 2), based on eigenvalue and scree plot review. Based on subscale composition, we labeled the four subscales as ‘Mobility and social engagement’, ‘Meeting family needs’, ‘Comfort with relationships’, and ‘General life satisfaction.’ Raykov’s *ρ* for each of the subscales ranged from 0.73–0.97, suggesting satisfactory or better internal consistency for each of the subscales. The correlations between the four subscales are presented in Table 3, and factor loadings of the subscales were moderate to strong, ranging from 0.36–0.60 (Table 2). The goodness-of-fit statistics for the higher-order factor model offer preliminary support for this model. The likelihood ratio test chi-square statistic was 0.46 (*p* = 0.7939). The root mean squared error of approximation (RMSEA), comparative fit index (CFI), and standardized root mean squared residual (SRMR) were 0.000 (90% CI 0.000–0.163), 1.000, and 0.022, respectively.

**Table 2:** Constituent items of the post-fistula repair reintegration instrument and lower and higher-order rotated factor loadings

Lower-order factors	Item^a^	Rotated factor loading
Mobility and social engagement *ρ = 0.86*	I am comfortable moving around my community.	0.8858
	I am comfortable moving around my house.	0.7039
	I freely engage in work that is necessary or important to me*.*	0.7033
	I am able to make longer trips.	0.5427
	I am satisfied with the role that I hold in my community.	0.5875
	I am able to participate in social and religious activities as I want.	0.5153
	I am satisfied with my capacity for work.	0.4432
	I am satisfied with my capacity to meet my needs and the needs of any dependents.	0.3997
	I am comfortable with myself in the company of others.	0.3460
Meeting family needs *ρ = 0.97*	I have a role in my family which meets my needs.	0.9443
	I have a role in my family which meets the needs of my family members.	0.8835
Comfort with relationships *ρ = 0.73*	In general, I am comfortable with my personal relationships.	0.8012
	In general, I am comfortable with my relationship with my husband/partner.	0.6647
	In general, I am comfortable with my relationship with friends/other family members.	0.5071
	In general, I am comfortable with my relationship with my children.	0.4152
General life satisfaction *ρ = 0.95*	In general, I am satisfied with myself.	0.9630
	In general, I am satisfied with my life.	0.9129
	In general, I am satisfied with my health.	0.7597
Higher-order factor	Lower-order factor	Rotated factor loading
Reintegration	Mobility and social engagement	0.6011
	Meeting family needs	0.5607
	Comfort with relationships	0.3550
	General life satisfaction	0.4139

^a^Response choices for all items were: always, mostly, sometimes, rarely, not at all

**Table 3:** Correlations between lower-order factors comprising the post-fistula repair reintegration instrument

	Mobility and social engagement	Meeting family needs	Comfort with relationships	General life satisfaction
Mobility and social engagement	1.0000			
Meeting family needs	0.4111	1.0000		
Comfort with relationships	0.2308	0.2436	1.0000	
General life satisfaction	0.3163	0.2341	0.9765	1.0000

The distribution of our standardized reintegration scale at baseline had mean 33.2 (SD 20.4) and median 29.0 (IQR 19.5–40.0). Construct validity was assessed by comparing participants’ scores on the standardized reintegration scale with certain concurrent characteristics and to their responses to other validated measures theoretically related to reintegration (Table 4). Across duration of living with fistula, the pattern of reintegration score was not significantly different for most categories compared to women living with fistula for <3 months; however, women who had lived with fistula the longest (5+ years) had significantly higher reintegration score. Reintegration score was patterned but did not significantly vary by severity of urinary incontinence, severity of fecal incontinence, a one-item measure of general health, and presence of or number of additional physical symptoms. Participant level of reintegration did correlate significantly with validated measures of quality of life (QOL), depression, self-esteem, experiences of stigma, and social support in the expected directions. Across QOL subscales, reintegration at baseline correlated significantly with the physical, psychological, and social subscales while the relationships between reintegration and the overall QOL subscale and the environmental QOL subscale suggested a similar trend but did not reach statistical significance. The mean increase in reintegration score associated with each one-point increase in QOL was 0.36 (95% CI 0.15, 0.56) for the physical subscale, 0.49 (95% CI 0.16, 0.82) for the psychological subscale, and 0.46 (0.25, 0.67) for the social subscale. The magnitude of association between depression symptoms and reintegration score was higher; for each one-point increase in depression symptoms, reintegration score significantly decreased by a mean of 16.06 points (95% CI -22.80, −9.32). Self-esteem was positively associated with reintegration score; each one-point increase in self-esteem was associated with a 1.67 increase in reintegration score (95% CI 0.58, 2.75). All stigma subscales were significantly and negatively associated with reintegration score; each one-point increase in stigma was associated with a decrease in reintegration score of 0.27 (95% CI -0.46, −0.08) for verbal abuse, 0.27 (95% CI -0.40, −0.14) for negative self-perception, 0.34 (−0.52, −0.17) for social isolation, and 0.36 (95% CI -0.63, −0.08) for fear of contagion. Social support was largely significantly and positively associated with reintegration score; each one-point increase in social support was associated with a mean increase in reintegration score of 0.92 (95% CI 0.44, 1.40) overall, 2.19 (95% CI 0.94, 3.45) for partner social support, and 1.33 (95% CI 0.33, 2.33) for family social support. The relationship between friend social support and reintegration score suggested a positive trend but did not meet our defined threshold for statistical significance.

**Table 4:** Assessment of criterion validity for the post-fistula repair reintegration instrument

	Reintegration score				
	β	95% CI	P		r^2^
Duration of Fistula					0.11
< 3 Months	REF	-	-	-	
3–12 Months	−2.82	−18.83	13.28	0.725	
1–2 Years	−5.07	34.29	24.15	0.729	
3–5 Years	11.13	−8.25	30.51	0.255	
≥ 5 Years	13.28	1.01	25.56	0.034	
Severity of Urinary Incontinence	−0.83	−2.4	0.74	0.294	0.02
Severity of Fecal Incontinence	−0.92	−2.86	1.02	0.346	0.02
General Health	−1.52	−15.24	12.21	0.826	0.00
Physical Symptoms					
Number of Symptoms	−2.31	−5.17	0.55	0.112	0.04
Any Symptoms versus None	−8.31	−21.87	5.25	0.225	0.03
Quality of Life					
Overall	0.42	−0.07	0.91	0.094	0.05
Physical	0.36	0.15	0.56	0.001	0.18
Psychological	0.49	0.16	0.82	0.004	0.14
Social	0.46	0.25	0.67	<0.001	0.25
Environmental	0.27	−0.08	0.62	0.124	0.04
Depressive Symptoms	−16.06	−22.80	−9.32	<0.001	0.28
Self-Esteem	1.67	0.58	2.75	0.003	0.14
Stigma					
Verbal Abuse	−0.27	−0.46	−0.08	0.007	0.12
Negative Self Perception	−0.27	−0.40	−0.14	<0.001	0.23
Social Isolation	−0.34	−0.52	−0.17	<0.001	0.21
Fear of Contagion	−0.36	−0.63	−0.08	0.012	0.10
Social Support					
All	0.92	0.44	1.40	<0.001	0.20
Partner	2.19	0.94	3.45	0.001	0.17
Family	1.33	0.33	2.33	0.01	0.11
Friend	0.97	−0.23	2.17	0.11	0.04
Any Trauma	1.39	−4.08	6.86	0.614	0.00

The full scale evidenced high internal consistency reliability with Raykov’s *ρ* of 0.88. Temporal stability reliability was measured among the subset of participants available at two-weeks post-surgery (*n* = 38), comparing our baseline sample to data collected at two-weeks post-surgery prior to catheter removal and hospital discharge. Correlation of reintegration score between the two time points was high (*r* = 0.97) and statistically significant (*p* < 0.001).

## Discussion

This study is the first to develop and validate a measure of post-surgical reintegration following obstetric fistula surgery that can be used to monitor patient outcomes and evaluate intervention programming. Our preliminary validation of the instrument among a small cohort of Ugandan women suggests that the instrument has high internal consistency and temporal reliability, and excellent validity.

The results of our exploratory factor analysis revealed that our primary construct of reintegration is a higher-order multi-dimensional construct, made up of four subscales. These subscales, titled ‘mobility and social engagement’, ‘meeting family needs’, ‘quality of relationships’, and ‘general life satisfaction’, all fall under the higher order construct of reintegration. Our overall reliability statistic was quite high at *ρ* = 0.88; however, reliability varied across the four subscales from *ρ* = 0.97 for ‘meeting family needs’ to *ρ* = 0.73 for ‘comfort with ‘relationships’. The subscale ‘comfort with relationships’ was lower than the others and therefore merits further study.

As anticipated, our reintegration measure was significantly correlated in the expected directions with validated scales measuring quality of life, depression, self-esteem, experiences of stigma, and social support. These measures assess constructs theoretically related to our construct of reintegration, and confirmation of the statistical relationship between them provides strong evidence for construct validity of our measure.

However, not all of the characteristics and measures that we assessed were related as expected to reintegration. We found no strong pattern in reintegration score across the category of how long women had lived with fistula; however, women who had lived with fistula for five or more years had a significantly higher reintegration score when compared with women who had lived with fistula for less than three months. This finding, combined with the observed coefficients for the other categories of time among women with unrepaired fistula suggests that women who live longer with the condition may develop coping strategies. Indeed, this is supported by literature on various coping strategies adopted by women with fistula that may have changed their reporting on our reintegration scale. Watt et al. describe religious coping strategies as nearly universal supports in helping a sample of Tanzanian women navigate living with fistula [60]. Prayer and refuge in church or with religious figures was also cited as important for coping with the condition among Ugandan women by Kabayambi et al. [61]. However, less positive coping behaviors are also described in the literature and include self-isolation and adjustment of fluid consumption to mediate incontinence symptoms [60, 62]. We were also surprised to note no significant relationship between reintegration score and measures of severity of urinary or fecal incontinence, respectively. While the coefficients for these relationship were in the anticipated inverse direction as literature suggests that women with persistent incontinence continue to report lower quality of life and social status [7, 19, 20, 27], it is likely that this lack of effect was due to the timing of scale validation activities at baseline where there was less variability in severity of urinary incontinence, and the fact that less than 1 % of our sample presented with fecal incontinence.

While this validation of our post-surgical reintegration measure is promising, it must be considered preliminary due to several limitations. First, our validation sample consisted of only 60 individuals; this sample size is low, particularly given the number of items included within our measure [63]. Second, our preliminary validation activities took advantage of the largest sample size from our longitudinal sample, the baseline measure, at which point women had not yet returned to their families and communities post-surgery. While our conceptualization of reintegration as a condition-specific global functional status should permit assessment at a continuum of time points, further validation should be conducted with data collected following women’s return to their communities. Third, while each item included in the reintegration measure meaningfully loaded onto one of the four lower-order factors, for some items the loading pattern was more clear-cut than others (e.g., satisfied with capacity for work, satisfied with capacity to meet own needs and needs of any dependents, satisfied with community role, and comfort with self in presence of others). These items were maintained and allocated to the factor they loaded most highly on. Future research with larger samples should be conducted to tease apart which factors these items affiliated with most strongly. Finally, both our formative research for measure development and measure validation was conducted among women comprising the patient population for Mulago Hospital in Kampala, Uganda, and our results may not be generalizable to other cultural contexts. Further exploration of the construct of reintegration following fistula repair and the measurement instrument should be conducted in larger and culturally distinct populations.

## Conclusion

The post-fistula repair reintegration instrument is the first standardized measurement instrument to evaluate women’s reintegration with family, community, and life following surgical repair of obstetric fistula. As more women undergo surgical treatment for obstetric fistula, attention to the post-repair period is imperative, particularly given preliminary data suggesting that a certain proportion of women experience persistent physical and psychosocial morbidities that may be addressed by a continuum of care orientation. While further validation work will improve our understanding of this instrument and its utility across different cultural contexts, this work represents a first step towards improving the measurement of post-surgical reintegration success and has important implications for improving the evidence base of the epidemiology of post-surgical reintegration among women affected by fistula as well as the development and evaluation of fistula programming.

## Appendix

**Table 5:** Initial standardized reintegration scale items and scoring instructions

Number	Item
1.	I am comfortable moving around my house.
2.	I am comfortable moving around my community.
3.	I am able to make longer trips.
4.	I am comfortable with how my self-care needs are met *(*e.g.*, dressing, feeding, toileting, bathing)*
5.	I freely engage in work that is necessary or important to me *(such as paid or voluntary work, housework or studying,* etc.*)*
6.	I am satisfied with my capacity for work
7.	I am satisfied with my capacity to meet my needs and the needs of any dependents.
8.	I am able to participate in social and religious activities as I want to *(*e.g.*, attending church/mosque services, prayer, community functions/parties)*
9.	I am comfortable socializing with family, friends and/or acquaintances
10.	I have a role in my family which meets my needs *(Family means people with whom you live and/or relatives with whom you don’t live but see on a regular basis)*
11.	I have a role in my family which meets the needs of my family members *(Family means people with whom you live and/or relatives with whom you don’t live but see on a regular basis)*
12.	I am satisfied with the role that I hold in my community.
13.	In general, I am comfortable with my personal relationships.
14.	In general, I am comfortable with my relationship with my husband/partner.
15.	In general, I am comfortable with my relationship with my children.
16.	In general, I am comfortable with my relationship with friends/other family members.
17.	In general, I am comfortable with myself in the company of others.
18.	During the past month, I have often felt down, depressed or hopeless.
19.	In general, I feel hope for the future.
20.	In general, I am satisfied with myself.
21.	In general, I am satisfied with my health.
22.	In general, I am satisfied with my life.

Instructions to respondent: The following questions you about your daily activities and feelings. Please indicate how frequently each statement describes you or your situation. (Code: 1 = Always, 2 = Mostly, 3 = Sometimes, 4 = Rarely and 5 = Not at all; indicate NA where relevant)

## Abbreviations

OF: Obstetric fistula
QOL: Quality of life
